# The LysR-type transcriptional regulator STY2660 is involved in outer membrane protein synthesis, bile resistance and motility in *Salmonella enterica* serovar Typhi

**DOI:** 10.3389/fmicb.2025.1554102

**Published:** 2025-02-26

**Authors:** Yitzel Gama-Martínez, Victor M. Hernández, Sergio Encarnación-Guevara, Ángel Martínez-Batallar, Ismael Hernández-Lucas

**Affiliations:** ^1^Departamento de Microbiologia Molecular, Instituto de Biotecnologia, Universidad Nacional Autonoma de Mexico, Cuernavaca, Mexico; ^2^Programa de Genomica Funcional de Procariotes, Centro de Ciencias Genomicas, Universidad Nacional Autonoma de Mexico, Cuernavaca, Mexico

**Keywords:** *serovar* Typhi, LysR regulator, bile resistance, motility, outer membrane protein

## Abstract

*Salmonella enterica* serovar Typhi, the etiological agent of Typhoid fever in humans, contains 44 LysR-type transcriptional regulators (LTTRs), most of which are annotated as hypothetical proteins whose roles are not yet described. In this work we demonstrated by mutants, growth evaluation in bile salts, transcriptional fusions, EMSAs, outer membrane protein profiles and motility assays, that the *S*. Typhi LTTR STY2660 is involved in two regulatory networks: FNR-STY2660-OmpR-OmpC for porin synthesis and bile resistance and FNR-STY2660-OmpR-FliD for motility. Thus, the LTTR STY2660 is able to establish genetic communication with master regulatory proteins to promote and efficiently respond to adverse conditions present in the host.

## 1 Introduction

Typhoid fever is caused by *Salmonella enterica* serovar Typhi (*S*. Typhi), a Gram-negative, rod-shaped bacterium of the *Enterobacteriaceae* family. To establish an infection, *S*. Typhi moves through the gastrointestinal tract and invades the spleen, liver, and gallbladder. In the latter compartment the pathogen survives in the presence of bile salts. For an efficient infection this pathogen utilizes a genetic arsenal that includes LysR-type transcriptional regulators (LTTRs).

LTTRs are DNA-binding proteins involved in the regulation of genes for porin synthesis, bile resistance, motility, host colonization, chemotaxis, and pathogenesis, among many other biological processes (Mayo-Pérez et al., 2023). Although LTTRs were initially described as regulators of its divergent gene, recent studies have demonstrated that these regulators are also able to control genetic elements located elsewhere in the genome. LTTRs are constituted by 300–350 amino acids and have a conserved structure that includes a DNA-binding domain (DBD) at the N-terminus connected by a long linker helix (LH) to a C-terminal binding domain, also called the regulatory domain (RD). The DBD interacts with the promoter and a co-inducer (glycine, acetolactate, D-Glutamate, among others) binds to the RD for transcriptional repression or activation of the target genes. Both domains are essential for the activity of LTTRs (Tropel and van der Meer, 2004; Guadarrama et al., 2014).

While LTTRs are present in eukaryoties and archaea, they are highly represented in bacteria (Mayo-Pérez et al., 2023), including *S*. Typhi, that contains 44 LTTRs in its genome. Previously we described the role of the LTTRs, LeuO and LtrR in *S*. Typhi pathogenesis (Hernández-Lucas et al., 2008; Rebollar-Flores et al., 2020; Villarreal et al., 2014). However, most of LTTRs present in *S*. Typhi genome are annotated as hypothetical proteins and their roles are not described. Hence, the putative LTTR STY2660 (WP_001109834.1) was selected for study since is widely conserved in the *Salmonella* genus and is divergent to a bile acid: sodium symporter, suggesting that STY2660 have a role in the resistance to bile salts. Thus, in this work we show that the LTTR STY2660 is fundamental in the regulation of porins, bile resistance and motility in *S*. Typhi.

## 2 Materials and methods

### 2.1 Bacterial strains, plasmids, and culture conditions

The bacterial strains and plasmids used in this work are listed in Supplementary Table S1. *Salmonella* Typhi IMSS-1 (Puente et al., 1987) and *Escherichia coli* strains were grown aerobically at 37°C in LB (10 g tryptone, 5 g yeast extract, and 10 g NaCl per liter) or N-MM media (0.37 g KCl, 0.99 g (NH_4_)_2_SO_4_, 0.087 g K_2_SO_4_, 0.14 g KH_2_PO_4_, 0.019 g MgCl_2_, 1 g casamino acids, 5 mL glycerol [100%], and 15.75 g of Tris–HCl (pH 7.5) per liter) (Deiwick et al., 1999). When required, the following antibiotics were added: kanamycin (Km), 30 μg ml^−1^; tetracycline (Tc), 12 μg ml^−1^, ampicillin (Ap), 200 μg ml^−1^.

### 2.2 Growth evaluation in 7.5% Ox bile and in 5% sodium deoxycholate

*S*. Typhi wild-type and mutant strains were grown for 24 h on LB plates at 37°C. A bacterial colony was inoculated in liquid LB broth (5 mL) and grown for 16 h at 37°C, 200 rpm. This culture was used to inoculate 50 mL of LB broth supplemented with 7.5% Ox bile (Sigma Chemical, St. Louis, MO) or 5% of deoxycholate (DOC) to an initial OD_595_ nm of 0.02. The cultures were incubated at 37°C, 200 rpm for 12 h with OD_595_ measurements being taken every 2 h.

### 2.3 DNA manipulations

Plasmid and genomic DNA isolations were carried out according to Sambrook et al. (1989). Primers for PCR amplifications were provided by the Oligonucleotide Synthesis Facility at our Institute (Supplementary Table S1). Restriction enzymes, ligase, nucleotides, and polymerases were acquired from New England Biolabs, Invitrogen, or Thermo Scientific. For sequencing, double-stranded DNA was purified with the Zyppy Plasmid Miniprep Kit (Zymo Research) and sequenced with an automatic Perkin Elmer/Applied Biosystems 377-18 system.

### 2.4 Construction of serovar Typhi mutants

The *S*. Typhi mutants were obtained by the one-step non-polar mutagenesis procedure (Datsenko and Wanner, 2000). The target gene was replaced with selectable antibiotic resistance gene markers, flanked with FRT sites. The resistance cassette was removed using the pCP20 (FLP helper plasmid). Each mutation was further characterized by sequencing to verify the authenticity of the deletion.

### 2.5 Construction of transcriptional reporter fusions

For transcriptional *cat* constructs, oligonucleotides (see Supplementary Table S1) were designed to amplify DNA fragments of different lengths from the LTTR STY2660, *fliC* (WP_000079784.1) *and fliD* (WP_000146805.1) regulatory regions. PCR products were double-digested with *Bam*HI-*Kpn*I and ligated into pKK232-8 or pKK232-9 (Supplementary Table S1), which contain the promoterless *cat* gene. All constructs were sequenced to verify the correct DNA sequence of the PCR fragments.

### 2.6 CAT assays

To determine the expression of the *cat* reporter gene mediated by the *S*. Typhi promoters, chloramphenicol acetyltransferase (CAT) assays were performed according to a previously published protocol (Martínez-Laguna et al., 1999). Briefly, *S*. Typhi strains harboring the reporters were grown in N-MM or LB supplemented either with 7.5% Ox bile or 5% DOC to an optical density (OD_595 nm_) of 1, and supplemented when required with Ap, Km, and 0.05 mM or 1 mM IPTG. Cells were harvested, centrifuged, washed with 0.8 mL of TDTT buffer (50 mM Tris–HCl, 30 μM _DL_-dithiothreitol, pH 7.8), resuspended in 0.6 mL of TDTT, and sonicated on ice for 10s intervals with 10s rest periods until the extract was clear (3 min). The homogenate was centrifuged at 12,000 g for 15 min at 4°C, and the supernatant used for activity measurement. For CAT assays, 5 μL of each extract were added in duplicate to a 96-well enzyme-linked immunosorbent assay (ELISA) plate, followed by the addition of 0.2 mL of a reaction mixture containing 1 mM DTNB [5,5′-dithiobis (2-nitrobenzoic acid)], 0.1 mM acetyl-coenzyme A (acetyl-CoA), and 0.1 mM chloramphenicol in 0.1 M Tris–HCl, pH 7.8. The absorbance was measured at 412 nm every 5 s for 5 min using a Ceres 900 scanning auto reader and microplate workstation. The protein concentration of the cell extracts was obtained using the bicinchoninic acid (BCA) protein assay reagent (Pierce). Protein values and the mean rate of product formation by CAT were used to determine CAT specific activity as μmol min^−1^ mg^−1^ protein. The results presented below are the means of three independent experiments with two technical replicates each experiment.

### 2.7 Plasmid construction and purification of STY2660 and FNR proteins

The STY2660 coding region (927 bp) was amplified by PCR, digested with *Nco*I and *Bam*HI, and cloned into the pFM*Trc12* vector, downstream of an IPTG inducible promoter resulting in plasmid pFM*Trc*12-STY2660.

The *S*. Typhi LTTR STY2660 and the *fnr* (WP_000611917.1) (753 bp) coding regions were amplified by PCR using specific primers: pMAL-c2X STY2660 *Bam*HI-F, pMAL-c2X STY2660 *Hind*III-R for STY2660, and pMAL-c2X *fnr Bam*HI-F, pMAL-c2X *fnr Hind*III-R for *fnr*. PCR products were digested with the corresponding restriction enzymes and ligated into the IPTG-inducible vector pMAL-c2X (New England Biolabs) to obtain the maltose binding protein-STY2660 construction (pMAL-c2X MBP-STY2660) and maltose binding protein-FNR construction (pMAL-c2X MBP-FNR). The recombinant plasmids were sequenced to verify the correct DNA sequence of the PCR fragments.

For purification, *E. coli* BL21 cells harboring independently the pMAL-c2X MBP-STY2660 and pMAL-c2X MBP-FNR were grown aerobically at 37°C to the mid-logarithmic phase (0.6 OD_595nm_) in 1 L of LB medium, then induced for 4 h with 1 mM IPTG to express the fusion proteins MBP-STY2660 and MBP-FNR. Cells were collected by centrifugation (5,000 g, 10 min at 4°C), resuspended in iced column buffer (200 mM Tris–HCl [pH 7.5], 200 mM NaCl, 1 mM EDTA, and 10 mM β-mercaptoethanol) and disrupted by French press. The suspension was centrifuged at 13,751 g for 10 min at 4°C to remove cell debris and the supernatant was incubated for 30 min with an amylose column (New England Biolabs) equilibrated with column buffer. The column was then washed with 5 volumes of column buffer, 1 volume with column buffer containing 0.1 mM maltose (Sigma), and finally with 5 volumes of column buffer. MBP-STY2660 and MBP-FNR proteins were eluted with 1 volume of column buffer containing 10 mM maltose (Sigma). The fractions were resolved by SDS-PAGE. Finally, the purified proteins were concentrated to 1 mL in dialysis buffer (40 mM Tris, 200 mM KCl and 20% glycerol, pH 7.4) using an Amicon Ultra 30 K device (Merck Millipore) at 2,500 g for 20 min. The protein concentration was determined by the Bradford method. Aliquots of the purified proteins were visualized in acrylamide gels and stored at −70°C.

### 2.8 Gel electrophoretic mobility shift assay

For non-radioactive EMSAs the DNA fragments of STY2660 (−395 + 118) or *ompR* (−383 + 169) were amplified by PCR using the primers described in Supplementary Table S1. Each DNA probe independently was mixed with increasing concentrations of purified proteins in the presence of 10× MBP-FNR (20 mM sodium phosphate buffer pH 8, 5% glycerol, 0.1 mg ml^−1^ BSA and 1 mM DTT) or 10× MBP-STY2660 binding buffer (20 mM sodium phosphate buffer pH 6.8, 5% glycerol, 0.1 mg ml^−1^ BSA and 1 mM DTT). Independent mixtures of the MBP-STY2660 and MBP-FNR proteins with the corresponding DNA fragments were incubated for 20 min at 37°C. MBP-FNR samples were resolved on 6% w/v polyacrylamide gels with 65 mM sodium phosphate buffer run at room temperature in 20 mM sodium phosphate buffer (pH 8) at 70 V for 2 h. MBP-STY2660 samples were resolved on 6% w/v polyacrylamide gels with 65 mM of sodium phosphate buffer run at room temperature in 20 mM sodium phosphate buffer (pH 6.8) at 70 V for 4 h. The DNA–protein complexes were visualized by ethidium bromide staining.

### 2.9 Outer membrane protein isolation, purification and electrophoresis

Outer membrane proteins (OMPs) were isolated from *S*. Typhi strains grown in N-MM and LB to an OD_595_ of 1.0 according to Puente et al. (1995), with minor modifications as described below. Twenty-five milliliters of each culture were harvested and centrifuged at 5,000 g for 8 min. Cells were resuspended in 500 μL of 10 mM Na_2_HPO_4_ buffer, pH 7.2, and sonicated on ice until the suspensions were clear. Intact cells and debris were eliminated by centrifugation (15,000 g) for 2 min, the supernatants were transferred to clean microcentrifuge tubes and membrane fractions were pelleted by centrifugation at 12,000 g for 1 h at 4°C. Inner membrane proteins were solubilized by resuspension in 500 μL of 10 mM Na_2_HPO_4_ buffer, pH 7.2, containing 2% Triton X-100 for 30 min at 37°C. After incubation, the samples were centrifuged at 12,000 g for 1 h at 4°C. The remaining outer membrane insoluble fraction was washed with 500 μL of 10 mM Na_2_HPO_4_, pH 7.2, centrifuged at 12,000 g for 1 h at 4°C and finally resuspended in 50 μL 1× PBS, pH 7.4. OMP concentrations were determined by BCA assay (Thermo) and 15 μg of each sample was analyzed by SDS-12% polyacrylamide gel electrophoresis. OMPs gels were visualized by staining with Coomassie brilliant blue R-250.

### 2.10 Swimming assays

To evaluate the motility of *S*. Typhi IMSS-1, *S*. Typhi Δ*fnr, S*. Typhi ΔSTY2660 and *S*. Typhi Δ*ompR* they were grown aerobically at 37°C to the mid-logarithmic phase (1 OD_595nm_) in 50 mL of LB medium. Five microliter of culture was spotted gently in the middle of a swim plate (LB, 0.3% bacteriological agar). The plates were incubated at 37°C for 12 h. The rates of migration from the point of inoculation, visible as a turbid zone, were measured at 12 h. The results are the means of at least 3 independent experiments.

## 3 Results

### 3.1 The serovar Typhi LTTR STY2660 is involved in Ox bile, sodium deoxycholate resistance and is induced specifically in the presence of the human bile salt DOC and in N-MM

**Figure 1:**
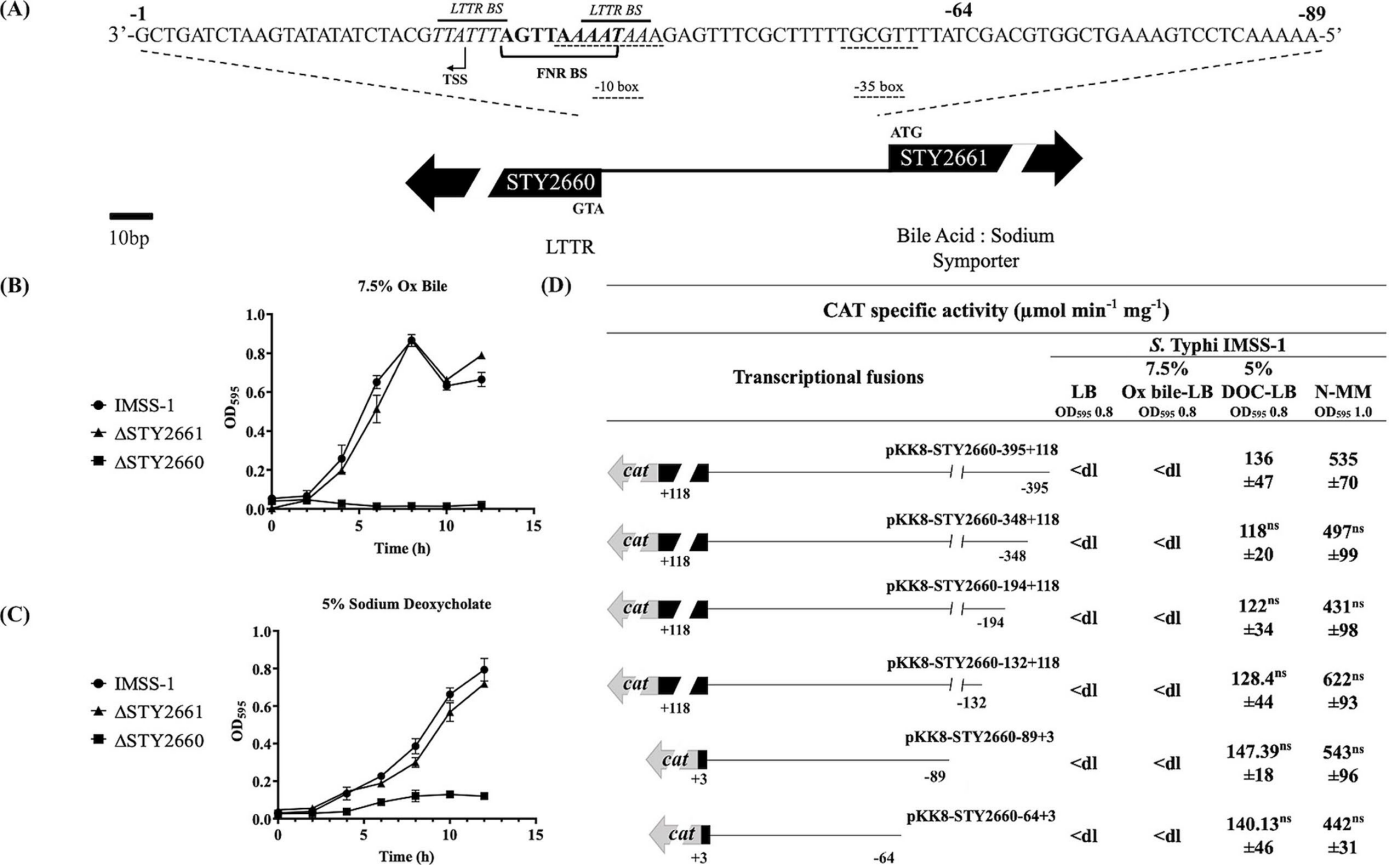
LTTR STY2660 is involved in Ox bile and DOC resistance and is induced by the human bile salt DOC and in N-MM. Genomic organization of the STY2660 and STY2661 genes. STY2660 intergenic regulatory region of 84 bp. The putative transcriptional start site is shown as a bent arrow. The putative −10 and −35 sites are indicated with doted lines. LTTR binding sites are indicated with italics. The FNR binding site is indicate with a bracket **(A)**. Growth curves of *S*. Typhi IMSS-1 (black circle), *S*. Typhi IMSS-1 ΔSTY2661 (black triangle) and *S*. Typhi IMSS-1 ΔSTY2660 (black square) in LB supplemented with Ox bile **(B)**, and DOC. **(C)** At least three independent experiments were performed, and representative data are shown. Transcriptional profile of STY2660 in *S*. Typhi IMSS-1 in LB supplemented with 7.5% of Ox bile, LB supplemented with 5% DOC and in N-MM. CAT specific activities were measured at an OD_595_ of 0.8 for Ox bile and DOC and 1.0 for N-MM **(D)**. The values are the means ± standard deviations for three independent experiments performed in duplicate. The *p* values were calculated with one-way Anova and Tukey’s test (ns, not significant). <dl (<detection limit) represents values between 0 and 10 CAT units.

Previously, we characterized LtrR and LeuO in *S*. Typhi, and in this study, we present a characterization of STY2660. This LTTR contains the characteristic DBD, LH, and RD domains of this family (Supplementary Figure S1). STY2660 is divergent to STY2661 (WP_000765569.1), annotated as a bile salt transporter (Figure 1A). Since diverse members of the LysR family regulate their divergent gene, we evaluated whether STY2660 and STY2661 play a role in bile resistance in *S*. Typhi. Thus, we deleted the STY2660 and the STY2661 genes in *S*. Typhi and assessed their role in bile resistance. Previously, we demonstrated that *S*. Typhi is able to grow in 7.5% Ox bile and in 5% of the human bile salt DOC (Villarreal et al., 2014). Growth curves in LB supplemented with Ox bile showed that STY2661 is not involved in bile resistance, since the corresponding mutated strain was able to grow like the *S*. Typhi IMSS-1 strain in LB supplemented with 7.5% Ox bile. In contrast, ΔSTY2660 is unable to grow in LB supplemented with 7.5% Ox bile, suggesting that this LTTR is involved in bile resistance (Figure 1B). We also evaluated resistance in LB supplemented with 5% DOC. The STY2661 mutant grew as well as the *S*. Typhi wild-type strain in the presence of 5% DOC, while the STY2660 mutant did not grow in the presence of DOC (Figure 1C). In contrast, the *S*. Typhi wild-type and the mutant strains display similar growth in LB medium (Supplementary Figure S2).

These results demonstrated that STY2660 is involved in bile and DOC resistance (Figures 1B,C). To evaluate whether Ox bile and DOC induce STY2660 genetic expression, a gene fusion collection containing different lengths of the STY2660 5′ intergenic regulatory region was constructed and then transferred into *S*. Typhi IMSS-1 and in the *S*. Typhi ΔSTY2660. Their expression was evaluated in the presence of Ox bile and DOC independently. The results showed that STY2660 was induced in the presence of DOC but not by Ox bile (Figure 1D), since the transcriptional expression of the fusions in the latter condition showed null CAT activity. Since the media that induced STY2660 expression was LB supplemented with 5% DOC, we evaluated STY2660 expression in unsupplemented LB media and found no transcriptional activity. To determine whether other media induce STY2660 expression, we evaluated N-MM minimal medium deficient in Mg and phosphate, which is reported to induce the expression of some virulence genes in *Salmonella* (Deiwick et al., 1999). We found that STY2660 expression was induced in this medium (Figure 1D).

The regulatory region involved in N-MM and DOC response correspond to 64 bp upstream of the STY2660 translational start site ATG, since the small fusion pKK8-STY2660-64 + 3, shows the same activity values as the larger fusion pKK8-STY2660-395 + 118 (Figure 1D). *In silico* analyzes of this 64 bp region raveled a putative transcription start site (TSS) located 26 bp upstream of the ATG translation start site, as well as a consensus sequence −10 and −35. This suggests that the minimal regulatory region of STY2660 lies within this 64 bp region (Figure 1A). In summary, STY2660 is fundamental in Ox bile resistance and is induced by the human bile salt DOC as well as by N-MM. Furthermore, the regulatory region involved in DOC and in N-MM induction lies within 64 bp upstream of the STY2660 ATG translational start codon in *S*. Typhi.

### 3.2 The LTTR STY2660 and FNR negatively regulate STY2660 genetic expression

To determine the regulators involved in the genetic control of STY2660 a bioinformatic analysis of its regulatory region was performed. The results showed the presence of two LTTR binding sites (5′AATAAA-N_5_-TTTATT3′) located −24 to −29 bp and −35 to −40 bp upstream of the STY2660 ATG translational start site and one FNR binding site (5′TTGAT3′), located at −29 to −33 bp upstream of the STY2660 translational start site. This localization suggests a negative role of both proteins in the genetic expression of STY2660, since the binding sites of both proteins overlap with the −10 TATA box of the STY2660 promoter region located at −33 to −41 bp upstream of the ATG (Figure 1A).

**Figure 2:**
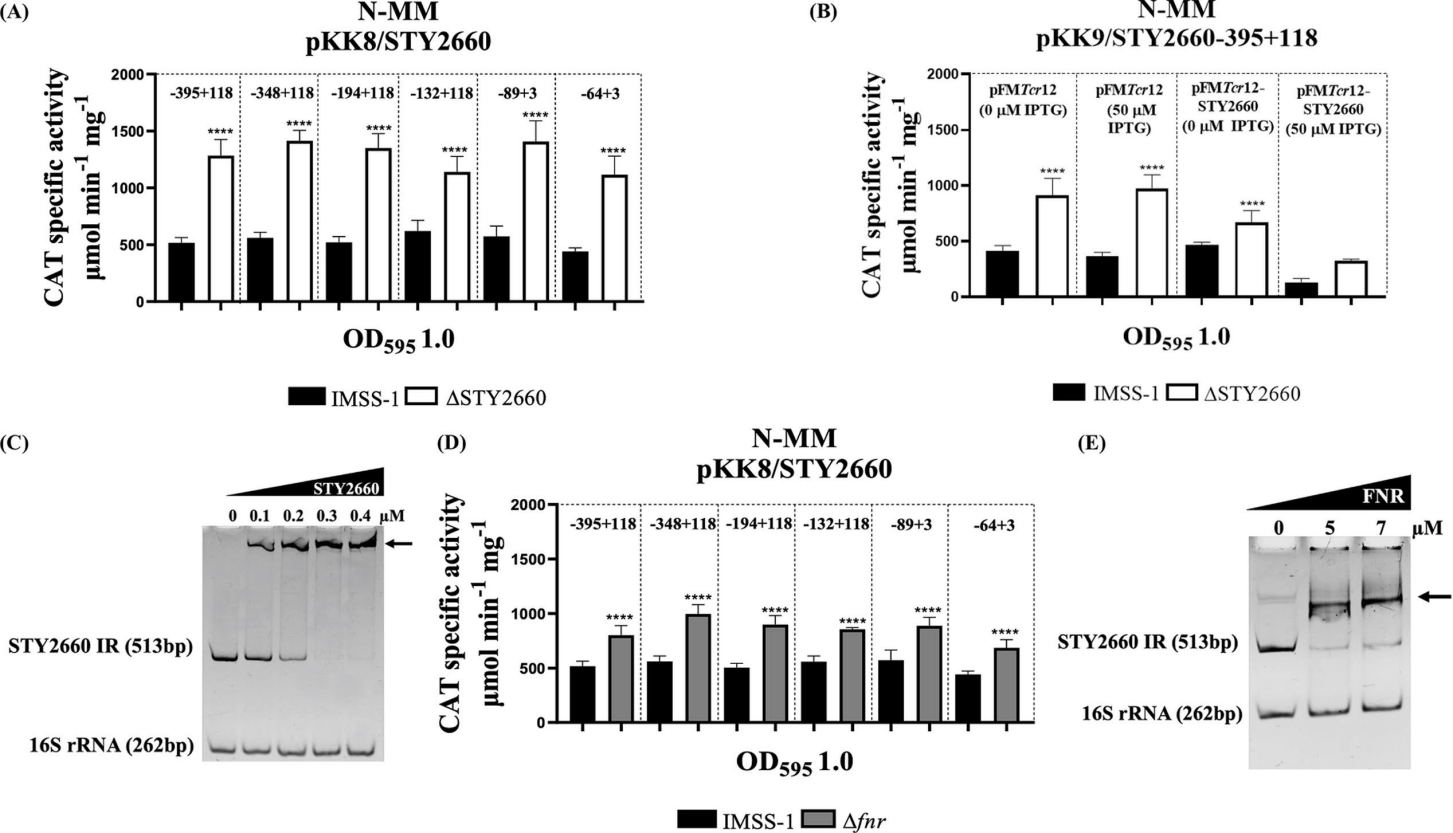
The LTTR STY2660 negatively and directly regulates its own transcriptional expression and is repressed by FNR. STY2660 transcriptional activities in *S*. Typhi IMSS-1 (black bars) and in *S*. Typhi ΔSTY2660 (white bars). The regions evaluated are indicated at the top of the graphs **(A)**. Transcriptional profile of STY2660 in *S*. Typhi IMSS-1 (black bars), *S*. Typhi IMSS-1 ΔSTY2660 (white bars) harboring pFM*Trc*12-STY2660 plasmid with or without 50 μM IPTG **(B)**. EMSAs of the MBP-STY2660 protein in the presence of the 5′ intergenic region of STY2660 (513 bp). The 16S rRNA (262 bp) was used as a negative control **(C)**. STY2660 transcriptional activities in *S*. Typhi IMSS-1 (black bars) and in *S*. Typhi Δ*fnr* (gray bars). The region evaluated is indicated at the top of the graph **(D).** EMSAs of the MBP-FNR protein in the presence of the 5′ intergenic region of STY2660 (513 bp). The 16S rRNA (262 bp) was used as a negative control **(E)**. Increasing concentrations of purified MBP-FNR or MBP-STY2660 were incubated with the corresponding DNA fragments. The EMSA experiments were resolved in 6% polyacrylamide gels and stained with ethidium bromide. Arrows indicate DNA–protein complex. The strains were grown in N-MM and CAT specific activity was measured at OD_595_ of 1.0. The values are the means ± standard deviations for three independent experiments performed in duplicate. The *p* values were calculated with one-way Anova and Tukey’s test (****p < 0.0001).

Since autoregulation is a common property among LTTRs we evaluated whether STY2660 repress its own transcriptional expression. Transcriptional expression of STY2660 in N-MM was increased approximately 58% in the *S*. Typhi ΔSTY2660 strain compared to *S*. Typhi IMSS-1 in all the fusions evaluated, indicating that STY2660 expression was repressed by its encoded product (Figure 2A). Furthermore, the complete STY2660 gene was cloned into the inducible IPTG plasmid, pFM*Trc*12. The resulting plasmid pFM*Trc*12-STY2660 and the empty vector (pFM*Trc*12) were transferred via electroporation into the *S*. Typhi IMSS-1, *S*. Typhi ΔSTY2660, with both strains also containing the pKK232-9/STY2660-395 + 118 reporter plasmid. The pFM*Trc*12-STY2660 was induced with 50 μM IPTG and the pKK232-9/STY2660-395 + 118 expression was reduced 68% in *S*. Typhi IMSS-1 and 69% in *S*. Typhi ΔSTY2660 compared with the expression results of non-induced plasmids pFM*Trc*12-STY2660 and pFM*Trc*12. The results indicate that the STY2660 protein inhibits its own transcriptional expression at the same levels in *S*. Typhi IMSS-1 and in *S*. Typhi ΔSTY2660. This result confirmed that STY2660 expression was repressed by its encoded product (Figure 2B). To evaluate whether STY2660 binds directly to its intergenic region, we performed EMSAs using the STY2660 protein and a 513 bp PCR product that included 118 bp of the STY2660 coding region, the complete 89 bp STY2660 5′ intergenic region and 306 bp upstream of its 5′ intergenic region. The formation of slow-migrating protein-DNA complexes demonstrated that this LTTR protein binds with high affinity to its regulatory region, concluding that STY2660 binds directly and autoregulates its transcriptional expression (Figure 2C).

Considering that the bioinformatics results also show the presence of a putative FNR binding site in the STY2660 regulatory region, we evaluated the expression of STY2660 in *S*. Typhi IMSS-1 and *S*. Typhi Δ*fnr*. The expression results in N-MM showed that FNR repress STY2660 expression, since the activity of this LTTR increased 44% in the *S*. Typhi Δ*fnr* compared with the *S*. Typhi IMSS-1 strain (Figure 2D). To evaluate whether FNR binds directly to the STY2660 regulatory region, we performed EMSAs of the FNR protein in the presence of the 5′ intergenic regions of STY2660 (513 bp) and demonstrated that the FNR protein binds to the STY2660 regulatory region, indicating that FNR directly represses STY2660 transcriptional expression (Figure 2E).

### 3.3 FNR directly regulates STY2660 and this LTTR interacts with *ompR* genetic region for *ompC* and *ompF* expression

Previously we reported that LtrR is involved in *ompR*-*ompC* transcriptional expression for the optimal growth of *S*. Typhi in the presence of Ox bile and DOC (Villarreal et al., 2014). Therefore, we evaluated whether FNR and the LTTR STY2660 regulate *ompR*-*ompC* expression for bile and DOC resistance.

**Figure 3:**
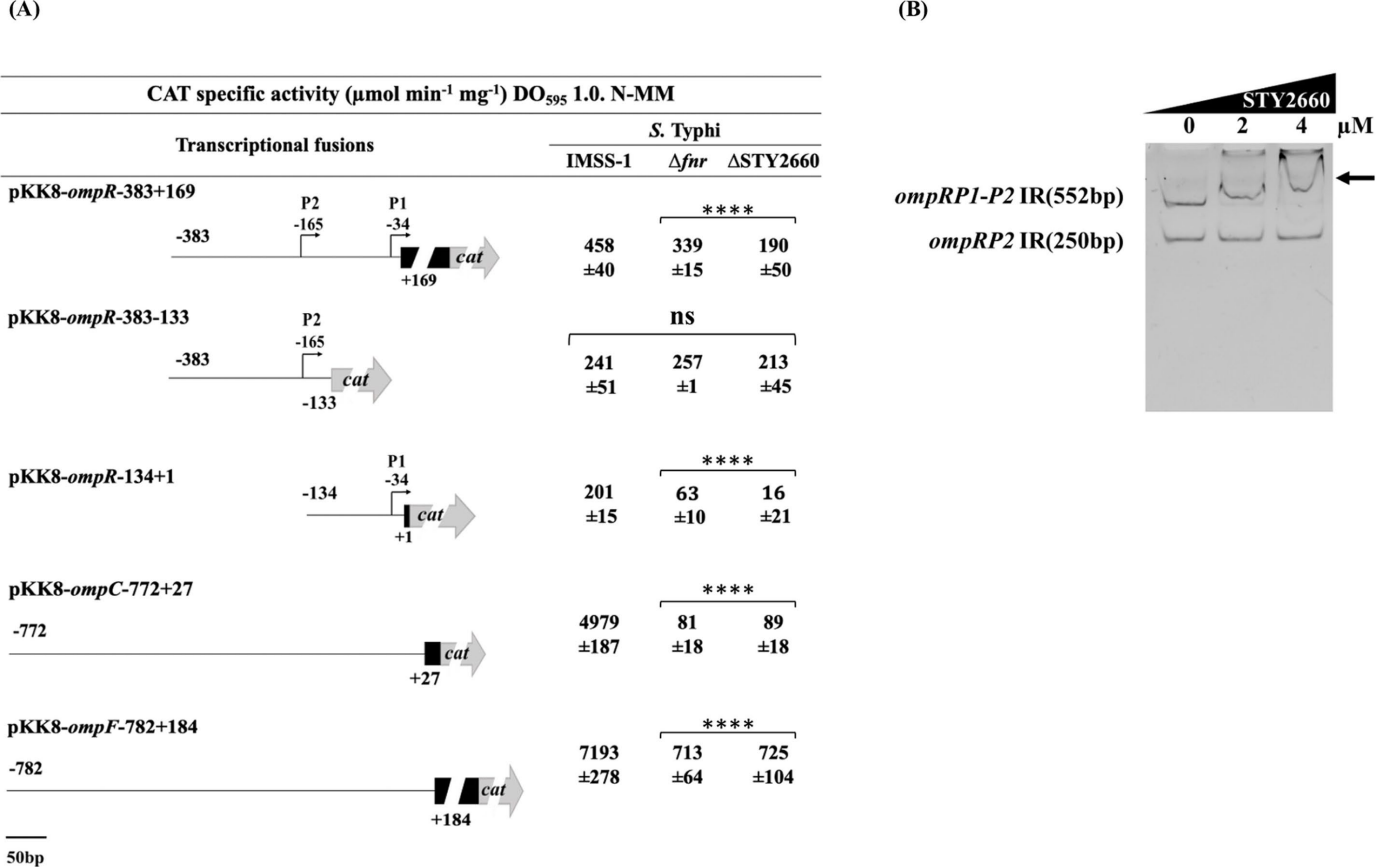
FNR and STY2660 regulate *ompR* expression and this LTTR interacts with this two-component regulator of OmpC and OmpF synthesis. Transcriptional expression of *ompR*, *ompC*, and *ompF* fusions in *S* Typhi IMSS-1, *S*. Typhi Δ*fnr* and *S*. Typhi ΔSTY2660. CAT activities were determined at an OD_595_ of 1.0 in N-MM. The values are the means standard deviations from three independent experiments performed in duplicate. The *p* values were calculated with one-way Anova and Tukey’s test (****p < 0.0001, ns, not s significant). **(A)** EMSAs of the MBP-STY2660 protein in presence of the 5′ intergenic region of *ompRP1-P2* (552 bp). The *ompR*P2 promoter (250 bp intergenic *ompR* region) that is not regulated by STY2660 was used as a negative control **(B)**. Increasing concentrations of purified MBP-STY2660 were incubated with the corresponding DNA fragments. The EMSA experiments were resolved in 6% polyacrylamide gels and stained with ethidium bromide. Arrows indicate DNA–protein complex.

To do this, an *ompR* fusion (pKK8/*ompR*-383 + 169) was introduced into *S*. Typhi IMSS-1, *S*. Typhi Δ*fnr* and in the *S*. Typhi ΔSTY2660 strains independently. The *ompR* expression results demonstrated that this global regulator is dependent on FNR and STY2660 since its transcriptional values were lower in both mutant strains compared with the *S*. Typhi IMSS-1 wild-type strain (Figure 3A). Previously we demonstrated that *ompR* contains two promoters, *ompR*P1 and *ompR*P2, with *ompR*P1 containing a LTTR binding site (Villarreal et al., 2014). Thus, we determined which *ompR* promoter is dependent on FNR and STY2660. The *ompR* fusions pKK8/*ompR*-134 + 1 containing the *ompR*P1 promoter and fusion pKK8/*ompR*-383-133 containing the *ompR*P2 promoter were introduced independently into *S*. Typhi Δ*fnr* and in the *S*. Typhi ΔSTY2660. The *ompR* transcriptional results demonstrated that the *ompR*P2 promoter is independent of FNR and STY2660, since its expression values in the corresponding mutant strains were similar to those in the *S*. Typhi wild-type strain. However, the *ompR*P1 promoter was dependent on both FNR and STY660, since its expression was lower in *S*. Typhi Δ*fnr* and *S*. Typhi ΔSTY2660 compared with *S*. Typhi IMSS-1 (Figure 3A).

Considering that OmpR regulates the major outer membrane porins OmpC and OmpF, we determined whether *ompC* and *ompF* were FNR and STY2660 dependent. An *ompC* fusion (pKK8/*ompC*-772 + 27) and *ompF* fusion (pKK8/*ompF*-782 + 184) were transformed into *S*. Typhi Δ*fnr* and *S*. Typhi ΔSTY2660 independently. The expression results showed that *ompC* and *ompF* are dependent on STY2660 and FNR, since the transcription of these two porin genes in the STY2660 showed a reduction of 98.2% for *ompC* and 89.9% for *ompF* compared with the wild-type *S*. Typhi strain. In the FNR mutant we found a reduction of 98.38% for *ompC* and 90% *ompF*, compared with the wild-type *S*. Typhi IMSS-1 (Figure 3A). Collectively these data demonstrate that FNR and STY2660 are essential for inducing *ompR* expression, which is crucial for *ompC* and *ompF* regulation.

The transcriptional results indicate that FNR directly regulates STY2660 and this LTTR modulates *ompR* expression for the transcription of *ompC* and *ompF*, suggesting the presence of the regulatory network *fnr*-STY2660-*ompR*-*ompC-ompF* in *S*. Typhi. To validate this hypothesis, EMSA experiments using the STY2660 protein and the *ompRP1*-*P2* (552 bp) and *ompRP2* (250 bp) promoters were performed and showed that STY2660 binds at *ompRP1*-*P2*. However, STY2660 is unable to interact with the *ompRP2* promoter region (Figure 3B). Thus, we defined that FNR interacts with the STY2660 promoter region and this LTTR interacts with *ompR* promoter region to regulate *ompC* and *ompF* expression, confirming the presence of an FNR-STY2660-OmpR regulatory network in *S*. Typhi.

### 3.4 FNR-STY2660-OmpR-OmpC regulatory network is fundamental for Ox bile and DOC resistance in serovar Typhi

**Figure 4:**
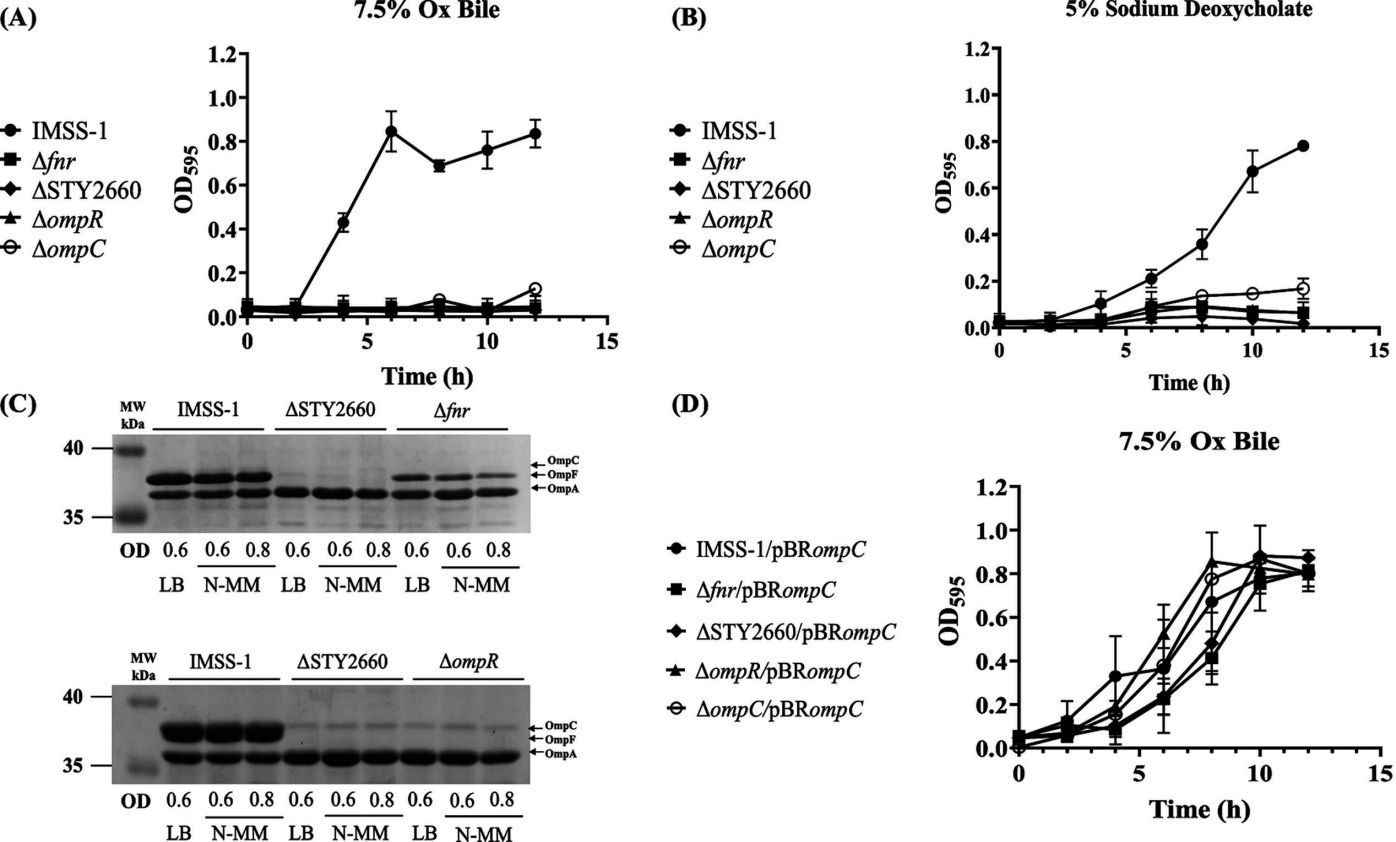
The genetic network FNR-STY2660-OmpR-OmpC is fundamental for the resistance of Ox bile in *S*. Typhi. Growth curves of *S*. Typhi wild-type strain and its derivatives Δ*fnr*, ΔSTY2660, Δ*ompR*, Δ*ompC* in LB supplemented with 7.5% Ox bile at 37°C **(A)** or 5% DOC at 37°C **(B)**. Electrophoretic pattern of Comassie brilliant blue-stained outer membrane protein preparations, separated by 0.1% SDS-15% PAGE from *S*. Typhi IMSS-1*, S*. Typhi ΔSTY2660, *S*. Typhi Δ*fnr* and *S*. Typhi Δ*ompR* grown in LB and N-MM at OD_595_ of 0.6 and 0.8. OmpC, OmpF, and OmpA are indicate with black arrows **(C)**. Growth curves of *S*. Typhi IMSS-1/pBR*ompC*, and their derivative complemented strains Δ*fnr*/pBR*ompC*, ΔSTY2660/pBR*ompC*, Δ*ompR*/pBR*ompC*, Δ*ompC*/pBR*ompC* in LB supplemented with 7.5% Ox bile at 37°C **(D)**. At least three independent experiments were performed, and representative data are shown.

Our transcriptional and DNA-protein interaction results demonstrated the presence of the FNR-STY2660-OmpR regulatory network. In Figures 1B,C we show that STY2660 is involved in bile resistance and DOC. Therefore, we evaluated whether all the genetic components of the FNR-STY2660-OmpR-OmpC-OmpF regulatory network are involved in bile and in DOC resistance by growth curves. The growth rate of independent mutants in Ox bile and DOC demonstrated that the single *fnr*, STY2660, *ompR* and *ompC* mutants were unable to growth in the presence 7.5% of Ox bile and 5% of DOC (Figures 4A,B). However, the *ompF* mutant grew like wild type strain IMSS-1 under these conditions, thus *ompF* is not involved in bile resistance (data not shown). This result agrees to previous data reported by our group showing the role of OmpC in bile and DOC resistance (Villarreal et al., 2014).

Previously we determined that the presence of OmpC is fundamental for bile resistance in *S*. Typhi (Villarreal et al., 2014). Thus, we evaluated the presence of this outer membrane protein in *S*. Typhi Δ*fnr*, *S*. Typhi ΔSTY2660 and *S*. Typhi Δ*ompR*. The porin profile showed that OmpC was absent in *S*. Typhi Δ*fnr*, in *S*. Typhi ΔSTY2660, OmpC and OmpF were not visualized. In *S*. Typhi Δ*ompR*, OmpC and OmpF showed null expression of these proteins (Figure 4C). These results are in agreement with the growth curve in LB supplemented with Ox bile, which showed that *S*. Typhi Δ*fnr*, *S*. Typhi ΔSTY2660, *S*. Typhi Δ*ompR* and *S*. Typhi Δ*ompC* mutants strains lost the ability to grow in Ox bile when the OmpC porin was absent (Figure 4A).

Complementation experiments with the wild type *ompC* gene of *S*. Typhi showed that the mutant strains complemented with *ompC* had wild type growth in LB supplemented with 7.5% of Ox bile (Figure 4D).

### 3.5 The FNR-STY2660-OmpR regulatory network is involved in swimming motility and in the regulation of the flagellum *fliC* and *fliD* genes

**Figure 5:**
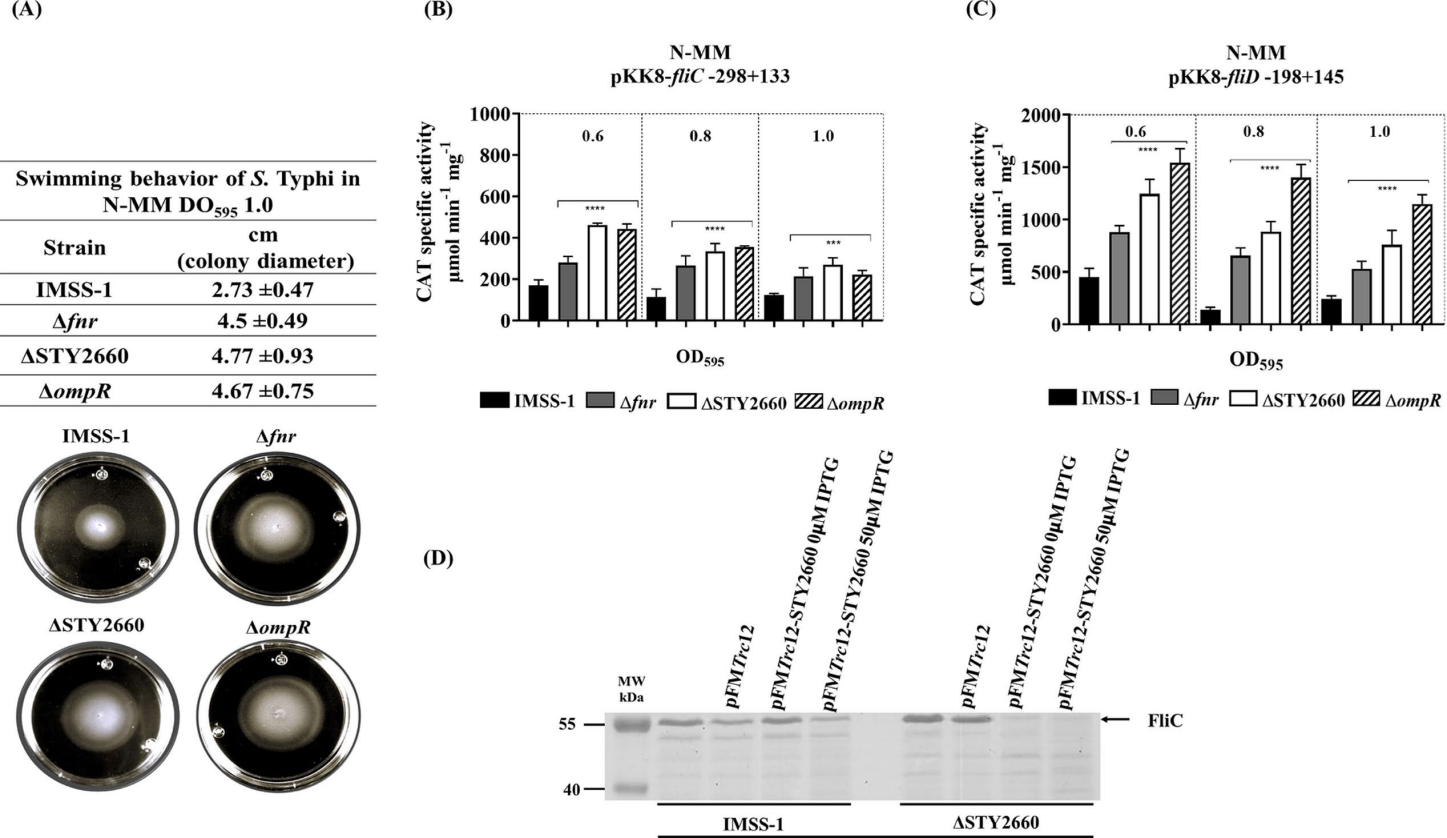
The FNR-STY2660-OmpR-FliC/FliD regulatory network is involved in swimming-motility. Swimming behavior of *S*. Typhi IMSS-1, *S*. Typhi Δ*fnr*, *S*. Typhi ΔSTY2660, and *S*. Typhi Δ*ompR* in swim medium N-MM (0.3%. agar). The migration rate and the images were obtained after 12 h of incubation at 37°C **(A)**. Transcriptional expression of *fliC* in *S* Typhi IMSS-1, *S*. Typhi Δ*fnr*, *S*. Typhi ΔSTY2660 and *S*. Typhi Δ*ompR*. CAT activities were determined at OD_595_ of 0.6, 0.8, and 1.0 in N-MM **(B)**. Transcriptional expression of *fliD* in *S* Typhi IMSS-1, *S*. Typhi Δ*fnr*, *S*. Typhi ΔSTY2660, and *S*. Typhi Δ*ompR*. CAT activities were determined at OD_595_ of 0.6, 0.8, and 1.0 in N-MM **(C)**. SDS PAGE 12%, showing that the pFM*Trc*12-STY2660 induced with IPTG repress FliC expression in the wild-type *S*. Typhi IMSS-1 and in *S*. Typhi ΔSTY2660. The proteins were visualized with Comassie blue **(D)**. The values are the means standard deviations from three independent experiments performed in duplicate. The *p* values were calculated with one-way Anova and Tukey’s test (****p < 0.0001; ***p < 0.001).

In this work we showed that FNR regulates STY2660 and this in turn controls *ompR* expression. It is well known that OmpR is involved in multiple biological process in different microorganism, for instance is induced in alkaline pH in *Vibrio cholera* (Kunkle et al., 2020). In the case of the *Salmonella* genus, OmpR is involved in swimming motility (Shin and park, 1995; Vidal et al., 1998). Thus, we evaluated whether FNR, and STY2660 via *ompR* regulation is involved in this process. Swimming assays showed that these genetic components are involved in this kind of motility, since the migration (increase in colony diameter) of *S*. Typhi Δ*fnr*, *S*. Typhi ΔSTY2660, and *S*. Typhi Δ*ompR* strains on N-MM plates were 64.8, 74.7, and 71.0% higher, respectively, than that of *S*. Typhi IMSS-1. This shows that the FNR-STY260-OmpR regulatory network represses motility, since the absence of these genetic components induces better migration rate in the corresponding mutant strains of *S*. Typhi (Figure 5A). Flagella are an essential component of swimming motility in bacteria. Thus, we evaluated whether *fliC* or *fliD*, which encode pivotal structural components of flagella, are regulated by FNR, STY2669 and OmpR in different ODs. The transcriptional results demonstrate that FNR, STY2660 and OmpR repress *fliC* and *fliD* expression, since in the *S*. Typhi IMSS-1 strain the transcriptional expression results were lower compared with those values obtained in the corresponding mutant strains (Figures 5B,C). We also evaluated whether STY2660 expression repressed FliC in *S*. Typhi IMSS-1 and in *S*. Typhi ΔSTY2660. FliC can be visualized in porin gels (Ballesté-Delpierre et al., 2016). With this system, we found that STY2660 expression in the wild-type strain and in the STY2660 mutant decreased expression of a 53.27 kDa protein (Figure 5D). To identify this protein the band were excised, trypsin-digested and analyzed by MALDI-TOF MS. The results showed that the STY2660-dependent protein correspond to FliC. The sequence coverage of this protein was 70% and the Mascot data base search algorithm revealed that the protein identified was identical to the corresponding proteins of several *S*. Typhi strains. Therefore, the phenotypic mobility assays and the transcriptional and protein analysis results demonstrated that the FNR-STY2660-OmpR regulatory network is involved in motility and in the regulation of fundamental genetic components of the flagellum.

## 4 Discussion

In this work we demonstrated that *S*. Typhi utilize multiple transcriptional regulators such as FNR, LTTR-STY2660 and OmpR to modulate outer membrane protein synthesis for bile resistance and flagellum expression for motility. In this regard FNR is known to be a global regulator which controls gene expression, facilitating bacterial adaptation to anaerobic conditions. FNR works as a regulator of genes involved in outer membrane synthesis, motility, and flagellar biosynthesis. Furthermore, FNR is also involved in porin synthesis in *E. coli* since are involved in the regulation of *ompW* (Xiao et al., 2016). In addition, we previously reported that FNR is involved in DOC resistance in *S*. Typhi (Olivar-Casique et al., 2022). The second component included in this study is the LTTR-STY2660. This transcriptional factor is a 308 amino-acid long and its structural analysis shows that the N-terminal domain from amino acids 5-64 encode a helix-turn-helix LysR motif and specifically the helix-turn-helix is from amino acids 20-39. The- C terminal domain encompass amino acids 89-292.

In this work we report that STY2660 is involved in the regulatory network FNR-STY2660-OmpR-OmpC for porin synthesis and resistance to bile and DOC. Previously we demonstrated that the LTTR LtrR regulates *ompR* and that this two-component regulator modulates *ompC* expression for bile resistance. Thus, two LTTRs STY660 and LtrR are involved in bile resistance through *ompR* control of OmpC synthesis. Regarding these data is relevant to mention that the signals and regulators that control LtrR and STY2660 genetic expression are different: LtrR is regulated by H-NS, LRP and pH, while STY2660 is under the control of FNR, and its expression increases in the presence of DOC. These LTTRs thus provide a genetic response to multiple signals and adverse conditions in the host, demonstrating the involvement of LTTRs in multiple regulatory networks utilized by *S*. Typhi to survive the presence of DOC and bile salts present in the small intestine and in the gallbladder.

The LTTR STY2660 of *S*. Typhi reported in this work is also involved in FNR-STY2660-OmpR-FliC/FliD regulatory network. The data demonstrated that FNR negatively regulates STY2660 and this LTTR represses FliC/FliD expression through *ompR* regulation. In this regard we propose that a main role of SY2660 in this regulatory network is to repress FliD-FliC and avoid flagellum formation, since this appendage is not necessary for survival in the presence of DOC (Olivar-Casique et al., 2022).

The data presented in this work indicate that exposure to DOC prompts *S*. Typhi to restructure and maintain cell wall integrity mediated by porins, while halting unnecessary processes such as flagella synthesis for motility. Both adaptations appear to be of critical importance for growth and survival in the presence of human bile salts and the LTTR-STY2660 is fundamental for these processes.

LTTR-STY2660 regulates *ompR* expression for porin synthesis and the role of this master two-component regulatory protein in this process is reported in several bacterial species (Villarreal et al., 2014; Yoshida et al., 2006). Furthermore, OmpR is involved in bile resistance (Doranga and Conway, 2023) and in the control of flagella production for motility (Hasan et al., 2023; Shin and park, 1995; Winter et al., 2015; Vidal et al., 1998). Thus, the function described in this work of the third component of the regulatory network FNR-STY2660-OmpR is in agreement with results observed in other bacteria.

The final targets of the FNR-STY2660-OmpR regulatory network are *ompC* and *fliC*-*fliD*. Previously, we described the role of OmpC in bacterial transformation and bile resistance and in this work the involvement of OmpC in bile resistance was confirmed. In the case of *fliC* encode for structural proteins that forms the flagellar filament and are essential for motility. *fliD* encode for the filament-cap protein of the flagellar apparatus is located at the distal end of the flagellum and plays a key role in the insertion of each flagellin protein at the growing tip of the flagellar filament. The flagellar filament is part of the bacterial flagellar motor thus, FliC and FliD are fundamental for correct flagella formation and bacterial motility (Arora et al., 2000) and in this work we showed that the FNR-STY2660-OmpR regulatory network represses *flic* and *fliD* transcriptional expression. These data are in agreement with the greater mobility of FNR, STY2660 or *ompR* mutants compared with the wild-type strain. Thus, the ability of this human pathogen to move efficiently in distant human compartments is a fundamental advantage that is provide by the FNR-STY2660-OmpR regulatory network.

**Figure 6:**
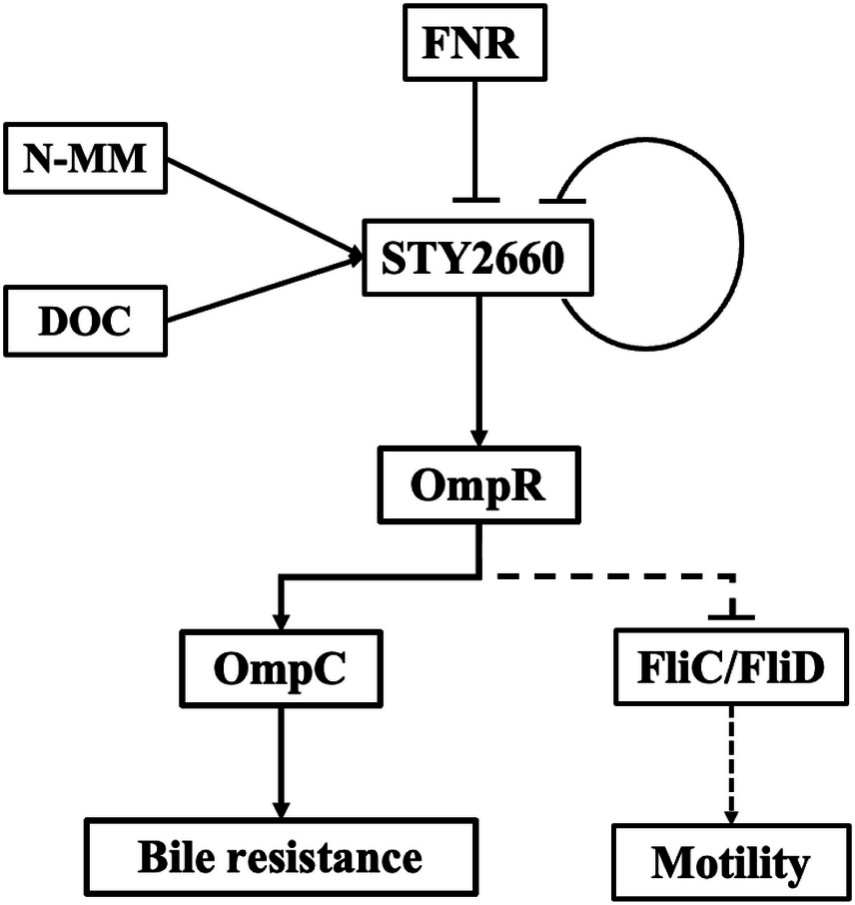
The FNR-STY2660-OmpR-OmpC-FliC/FliD regulatory network. FNR and STY2660 negatively regulate STY2660 expression. The N-MM and DOC positively regulate STY2660 expression for OmpR induction for porin synthesis-bile resistance and motility.

In summary, we describe here the functional relationships of FNR-LTTR-STY2660-OmpR in bile resistance and motility, two fundamental processes for efficient infection by *S*. Typhi. Thus, LTTRs are able to establish genetic communication with master regulatory proteins to promote an adequate cell response to human stress conditions. The signals, transcriptional regulators, dependent genes and the biological process in which The FNR-STY2660-OmpR-OmpC-FliC/FliD regulatory network is involved is depicted in Figure 6.

## Data Availability

The authors acknowledge that the data presented in this study must be deposited and made publicly available in an acceptable repository, prior to publication. Frontiers cannot accept a manuscript that does not adhere to our open data policies.
