# Supplementary figures and images for: The LysR-type transcriptional regulator STY2660 is involved in outer membrane protein synthesis, bile resistance and motility in Salmonella enterica serovar Typhi
Source: Front Microbiol. 2025 Feb 26;16:1554102. doi: 10.3389/fmicb.2025.1554102 (PMC11904634; doi:10.3389/fmicb.2025.1554102)

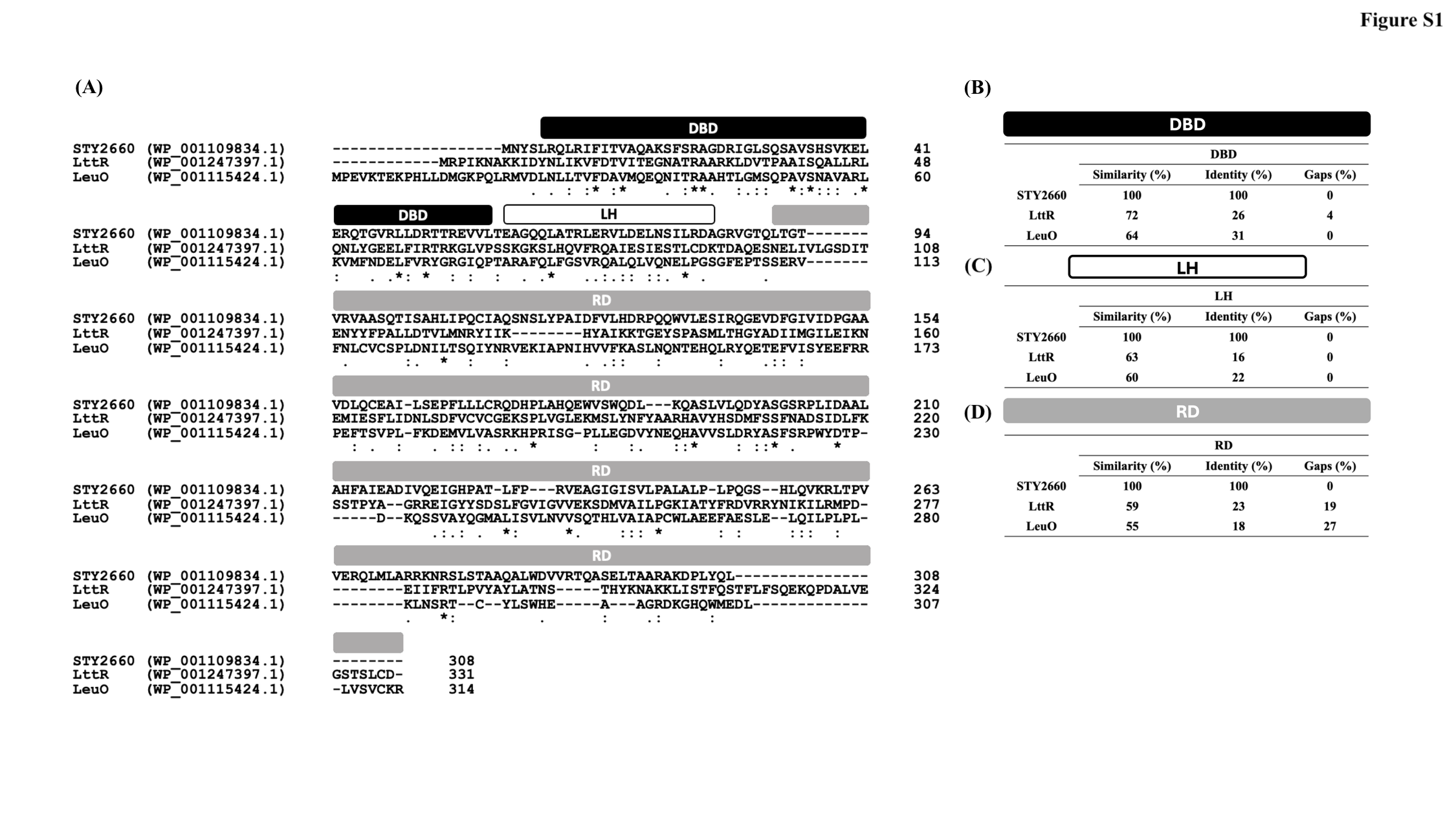

Supplement: Supplementary file 1 [file Image_1.tif]

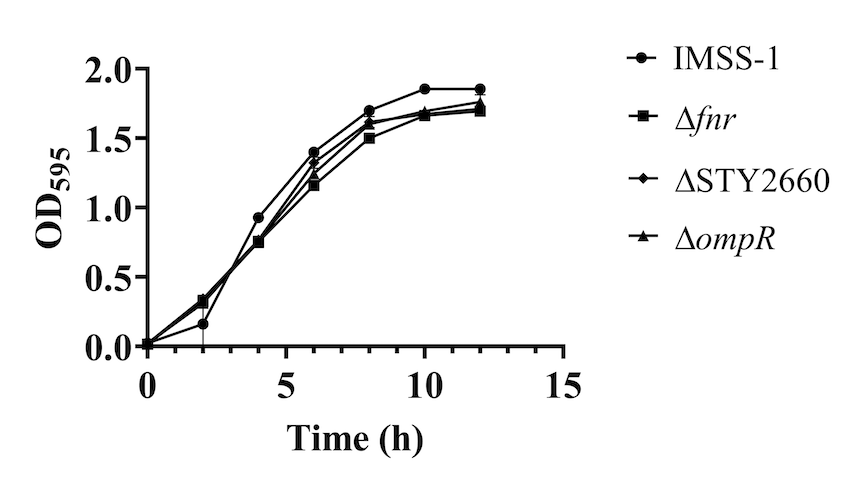

Supplement: Supplementary file 2 [file Image_2.tif]
