# Supplementary material for: The LysR-type transcriptional regulator STY2660 is involved in outer membrane protein synthesis, bile resistance and motility in Salmonella enterica serovar Typhi
Source: Front Microbiol. 2025 Feb 26;16:1554102. doi: 10.3389/fmicb.2025.1554102 (PMC11904634; doi:10.3389/fmicb.2025.1554102)
Supplement: Supplementary file 3 [file Data_Sheet_1.pdf]

SUPPLEMENTAL MATERIAL.

Fig. S1 Amino acid sequence alignment of STY2660, LtrR, and LeuO of *S*. Typi (A). The identical amino acids *, conservative substitutions :, semi-conservative substitutions ., are shown in figure. The identity and similarity of LTTRs characteristic domains, DNA-binding domain (DBD) **(B)**, linker helix (LH) **(C)**, and regulatory domain (RD) **(D)**.

Fig. S2 Growth curve of *S*. Typhi IMSS-1 and its derivative mutant strains in LB at 37°C. *S*. Typhi IMSS-1 (black circle), *S*. Typhi IMSS-1 Δ*fnr* (black square), *S*. Typhi IMSS-1 ΔSTY2660 (black diamond) and *S*. Typhi IMSS-1 Δ*ompR* (black triangle). The growth was monitored by OD_{595}. At least three independent experiments were performed, and representative data are shown.

TABLE S1. Bacterial strains, plasmids and primers used in this work.

**Strains**	**Genotype and/or relevant characteristics**	**Reference**
S. Typhi IMSS-1	*Salmonella enterica* serovar Typhi 9.12, d, serotype; Mexican reference clinical strain	Puente et al., 1987
S. Typhi IMSS-1+pFM*Trc12*	*S*. Typhi IMSS-1 containing the pFM*Trc12* plasmid. Ap^R	Hernández-Lucas et al., 2008
S. Typhi IMSS-1+ pFM*Trc12*-STY2660	*S*. Typhi IMSS-1 containing the pFM*Trc12*-STY2660 plasmid. Ap^R	This study
S. Typhi IMSS-1 ΔSTY2660	*S*. Typhi ΔSTY2660	This study

S. Typhi IMSS-1 ΔSTY2660+pFM*Trc12*	*S*. Typhi ΔSTY2660 containing the pFM*Trc12* plasmid. Ap^{R}	This study
S. Typhi IMSS-1 ΔSTY2660+ pFM*Trc12*-STY2660	*S*. Typhi ΔSTY2660 containing the pFM*Trc12*- STY2660 plasmid. Ap^{R}	This study
S. Typhi IMSS-1 Δ*fnr*	*S*. Typhi Δ*fnr* Km^{R}	Olivar-Casique et al., 2022
S. Typhi IMSS-1 Δ*ompR*	*S*. Typhi Δ*ompR* Km^{R}	Villarreal et al., 2014
E. coli DH5α	Φ80d/*lac*ZΔM15 Δ(*lac*ZYA-*arg*F) U169 *rec*A1*end*A1 *hsd*R17 ($r_k^- m_k^+$) *pho*A*sup*E44 λ- thi-*1 gyr*A96 *rel*A. Nal^{R}	Gibco BRL
E. coli BL21	Strain for expression of recombinant proteins, protease deficient	New England Biolabs
E. coli BL21+pMAL-c2X	*E. coli* BL21 containing the pMAL-c2X plasmid. Ap^{R}	This study
E. coli BL21+ pMAL-c2X-STY2660	*E. coli* BL21 containing the pMAL-c2X-STY2660 plasmid. Ap^{R}	This study
E. coli BL21+ pMAL-c2X-*fnr*	*E. coli* BL21 containing the pMAL-c2X-*fnr* plasmid. Ap^{R}	This study
**Plasmids**		
pFM*Trc*12	p*Trc99*A derivative containing the p15A1 origin of replication. Ap^{R}	Hernández-Lucas et al., 2008
pFM*Trc12*-STY2660	pFM*Trc12* derivative, containing the complete STY2660 gene (927 bp). Ap^{R}	This study
pMAL-c2X	Plasmid for constructing maltose binding protein (MBP) fusions, *lac* promoter. Ap^{R}	New England Biolabs
pMAL-c2X MBP-STY2660		This study

	pMAL-c2X derivative containing the STY2660 gene (927) without the TGA stop codon. Ap^R	
pMAL-c2X-*fnr*	pMAL-c2X derivative containing the *fnr* gene (753) without the TGA stop codon. Ap^R	This study
pKK232-8	pBR322 derivative containing a promoterless chloramphenicol acetyltransferase (*cat*) gene. Ap^R	Pharmacia LKB Biotechnology
pKK232-9	pKK232-8 derivative containing a promoterless chloramphenicol acetyltransferase (*cat*) gene. Km^R	Hernández-Lucas et al., 2008
pKK9-STY2660-395+118	pKK232-9 containing 395 bp upstream and 118 bp downstream of the STY2660 ATG start codon. Ap^R	This study
pKK8-STY2660-348+118	pKK232-8 containing 348 bp upstream and 118 bp downstream of the STY2660 ATG start codon. Ap^R	This study
pKK8-STY2660-194+118	pKK232-8 containing 194 bp upstream and 118 bp downstream of the STY2660 ATG start codon. Ap^R	This study
pKK8-STY2660-134+118		This study

	pKK232-8 containing 134 bp upstream and 118 bp downstream of the STY2660 ATG start codon. Ap^R	
pKK8-STY2660-89+3	pKK232-8 containing 89 bp upstream and 3 bp downstream of the STY2660 ATG start codon. Ap^R	This study
pKK8-STY2660-64+3	pKK232-8 containing 64 bp upstream and 3 bp downstream of the *ompR* ATG start codon. Ap^R	This study
pKK8-STY2660-63+3	pKK232-8 containing 64 bp upstream and 3 bp downstream of the STY2660 ATG start codon. Ap^R	This study
pKK8-*ompR*-383+169	pKK232-8 containing 383 bp upstream and 169 bp downstream of the *ompR* ATG start codon. Ap^R	This study
pKK8-*ompR*P1-134-1	pKK232-8 containing 134 bp upstream of the *ompR* ATG start codon. Ap^R	Villarreal et al., 2014
pKK8/*ompR*P2-383-133	pKK232-8 containing from 383 to 133 bp upstream of the *ompR* ATG start codon. Ap^R	Villarreal et al., 2014
pKK8-*ompF*-782+184	pKK232-8 containing 782 bp upstream and 184 bp downstream of the *ompF* ATG start codon. Ap^R	Villarreal et al., 2014
pKK9-*ompC*-772+27		Hernández-Lucas et al., 2008

	pKK232-9 containing 772 bp upstream and 27 bp downstream of the *ompC* ATG start codon. Km^R	
pKK8-*fliD*-198+145	pKK232-8 containing 198 bp upstream and 145 bp downstream of the *fliD* ATG start codon. Ap^R	This study
**Oligonucleotides**	**Sequence (5´-3´)**	
pMAL-c2x STY2660 *Bam*HI-F	CGG GAT CCA TGA ACT ATT CAC TGC GTC AAC TG	This study
pMAL-c2x STY2660 *Hind*III-R	GCC CAA GCT TTT ATA GCT GAT ACA GCG GAT CTT TCG	This study
PFM*Trc12* STY2660 *Nco*I-F	CAT GCC ATG GAC TAT TCA CTG CGT CAA CTG	This study
PFM*Trc12* STY2660 *Bam*HI-R	CGG GAT CCC CTG TTT TAA CAC ACT GCC CTG	This study
pMAL-c2x *fnr Bam*HI-F	CGG GAT CCA TCC CGG AAA AGC GAA TTA TAC GG	This study
pMAL-c2x-*fnr Hind*III-R	GCC CAA GCT TAA GCG ACG TTG CGG GTA TGA CCG GCG AG	This study
STY2660-395+118 *Kpn*I-R	GGG GTA CCC TTT TAC GCT ATG ACT GAC CGC	This study
STY2660-395+118 BamHI-F	CGG GAT CCA AAG CCG CTG TAG AGC ATA GGG	This study
STY2660-348+118 BamHI-F	CGG GAT CCC AAA CAG CAC GCC GAG GAC A	This study
STY2660-194+118 BamHI-F	CGG GAT CCT TCA ACG ACA GGC ACA AAG C	This study
STY2660-132+118 BamHI-F	CGG GAT CCT AAG TGT CAG CGT GAA AGG GTC	This study

STY2660-89+3 BamHI-F	CGG GAT CCA AAA ACT CCT GAA AGT CGG TGC AGC TAT TTT GCG TTT TTC GCT TTG AGA AAT AAA ATT GAT TTA TTG CAT CTA TAT ATG AAT CTA GTC GAT GGG TAC	This study
STY2660-89+3 KpnI-R	GGG GTA CCA TCG ACT AGA TTC ATA TAT AGA TGC AAT AAA TCA ATT TTA TTT CTC AAA GCG AAA AAC GCA AAA TAG CTG CAC CGA CTT TCA GGA GTT TTT G	This study
STY2660-64+3 BamHI-F	CGG GAT CCC TAT TTT GCG TTT TTC GCT TTG AGA AAT AAA ATT GAT TTA TTG CAT CTA TAT ATG AAT CTA GTC GAT GGG TAC	This study
STY2660-64+3 KpnI-R	GGG GTA CCA TCG ACT AGA TTC ATA TAT AGA TGC AAT AAA TCA ATT TTA TTT CTC AAA GCG AAA AAC GCA AAA TAG G	This study
fliD/-198+145 BamHI-F	CGG GAT CCC CGT AAC CCT TGT ATC GGC	This study
fliD/-198+145 KpnI- R	GGG TAC CCT GCC ATA GGC GGT TAG CTT	This study

The nucleotides underlined correspond to the *Bam*HI, *Hind*III, *Nco*I and *Kpn*I restriction sites.
